# Are Older Adults Up-to-Date With Cancer Screening and Vaccinations?

**Published:** 2005-06-15

**Authors:** Douglas Shenson, Julie Bolen, Laura Seeff, Donald Blackman, Mary Adams

**Affiliations:** The author is affiliated with Sickness Prevention Achieved through Regional Collaboration (SPARC), Lakeville, Conn; Centers for Disease Control and Prevention, Atlanta, Ga; Centers for Disease Control and Prevention, Atlanta, Ga; Centers for Disease Control and Prevention, Atlanta, Ga; On Target Health Data LLC, Hartford, Conn

## Abstract

**Introduction:**

Public health organizations in the United States emphasize the importance of providing routine screening for breast cancer, cervical cancer, and colorectal cancer, as well as vaccinations against influenza and pneumococcal disease among older adults. We report a composite measure of adults aged 50 years and older who receive recommended cancer screening services and vaccinations.

**Methods:**

We analyzed state data from the 2002 Behavioral Risk Factor Surveillance System, which included 105,860 respondents aged 50 and older. We created a composite measure that included colonoscopy or sigmoidoscopy within 10 years or a fecal occult blood test in the past year, an influenza vaccination in the past year, a Papanicolaou test within 3 years for women with an intact cervix, a mammogram, and for adults aged 65 and older, a pneumonia vaccination during their lifetime. We performed separate analyses for four age and sex groups: men aged 50 to 64, women aged 50 to 64, men aged 65 and older, and women aged 65 and older.

**Results:**

The percentage of each age and sex group that was up-to-date according to our composite measure ranged from 21.1% of women aged 50 to 64 (four tests) to 39.6% of men aged 65 and older (three tests). For each group, results varied by income, education, race/ethnicity, insurance status, and whether the respondent had a personal physician.

**Conclusion:**

These results suggest the need to improve the delivery of cancer screenings and vaccinations among adults aged 50 and older. We propose continued efforts to measure use of clinical preventive services.

## Introduction

*Healthy People 2010* is a national initiative that has set specific health and health care objectives (1). These performance targets include the delivery of adult clinical preventive services such as cancer screening and vaccinations. Studies of the effectiveness of these services have been reviewed by the U.S. Preventive Services Task Force (USPSTF) (2), and recommendations for providing the services have been developed by the *Guide to Community Preventive Services* (3). Both efforts emphasize the importance of providing routine screening for breast cancer, cervical cancer, and colorectal cancer as well as vaccinations against influenza and pneumococcal disease.  

The delivery rates of cancer screenings and adult vaccinations have typically been measured separately through population-based surveys, including the Behavioral Risk Factor Surveillance System (BRFSS) and the National Health Interview Survey (NHIS). Although this approach is well suited for assessing progress toward *Healthy People 2010* objectives, it does not measure the extent to which individuals receive the full complement of recommended clinical preventive services.

There is currently no index that combines data on the use of all recommended clinical preventive services among adults. In this article, we present a composite measure of cancer screening services and vaccinations obtained by adults aged 50 years and older as a first step toward assessing overall clinical preventive service delivery. We chose to begin our assessment by combining these two sets of interventions because they are universally recommended and data on their use in all 50 states and the District of Columbia are available through the 2002 BRFSS. Composite indices have long been used for the surveillance of childhood vaccinations (4).

Our approach aims to provide a more meaningful and practical measure of the state of clinical preventive service delivery. Such a comprehensive measure could also enhance the ability of health departments and community groups to assess disparities in delivering preventive services, to better gauge progress toward measurable objectives, and to identify best practices for achieving prevention goals.

## Methods

### Data

We used data from the 2002 BRFSS; the BRFSS is an ongoing state-based telephone survey of randomly selected adults and is coordinated by the Centers for Disease Control and Prevention (CDC). The survey collects data from noninstitutionalized adults aged 18 and older on health practices that are related to the leading causes of death and disability (5). We limited our analysis to data from adults aged 50 and older, including 105,860 respondents in 49 states and the District of Columbia. Illinois data are not reported because approximately half of the female respondents in that state were not asked questions about breast and cervical cancer screening. All results are based on weighted data that account for different probabilities of selection and are adjusted to the total adult population in each state by age and sex or age, race, and sex.

### Measures

We analyzed responses to the BRFSS core questions on the use of clinical preventive services recommended by the USPSTF for adults aged 50 and older: colorectal cancer screening, mammography, Papanicolaou (Pap) test, and influenza and pneumococcal vaccinations (Table 1). These questions were asked in all states and territories that participated in the 2002 BRFSS survey. All measures of preventive health services were dichotomized as yes or no based on receipt of the service according to schedules recommended by the USPSTF. The USPSTF recommends influenza vaccination for adults aged 50 and older (6); other agencies support this recommendation (7). We used a 10-year interval for endoscopy (colonoscopy or sigmoidoscopy) because the BRFSS question did not distinguish between the two interventions. The USPSTF does not recommend intervals for the use of sigmoidoscopy or colonoscopy; other national guidelines recommend a 5-year interval for sigmoidoscopy and a 10-year interval for colonoscopy (8-10). For all services, people who had never had the test or had the tests outside the designated schedule were included in the group who answered no. Our analysis excluded respondents with missing values except for respondents missing one colorectal cancer screening value. Because colorectal cancer screening recommendations involve receiving either endoscopy or fecal occult blood testing (FOBT), we did not exclude respondents with missing values for one test if they had the other test within the recommended interval.

From the separate measures for cancer screenings and vaccinations, we created a variable to measure whether a person had received all of these clinical preventive services as recommended by the USPSTF. Because the recommendations vary by age and sex, this measure, which we call being *up-to-date*, was determined separately for four age and sex groups. For men aged 50 to 64, the up-to-date measure included men who met the recommendation for colon cancer screening and influenza vaccination (two services). For women aged 50 to 64, the up-to-date measure included women who met the recommendations for colon cancer screening, breast cancer screening, cervical cancer screening, and influenza vaccination (four services). The up-to-date measure includes women with a hysterectomy among those who met recommendations for cervical cancer screening. Because a Pap test is not normally recommended for these women, it would be incorrect to classify them as not being up-to-date on cervical cancer screening. For adults aged 65 and older, the up-to-date measure included the cancer screening tests for their sex, plus an influenza vaccination and a pneumococcal vaccination (three services for men and five services for women). The number of tests required for being up-to-date was two for men aged 50 to 64, three for men aged 65 and older, four for women aged 50 to 64, and five for women aged 65 and older (colorectal, breast, and cervical cancer screening in addition to influenza and pneumonia vaccinations).

From responses to several questions on race and ethnicity that permitted respondents to indicate more than one race, we created five groups: white (limited to non-Hispanic whites), black (limited to non-Hispanic blacks), Hispanic of any race, Asian or Pacific Islander, and American Indian or Alaska Native. Level of education was recoded from multiple responses into four categories: less than high school, high school graduate or general equivalency diploma (GED), some college, and college graduate. Health insurance status was determined by the response to a single question and coded yes or no. Data on household income were coded into four groups: less than $25,000, $25,000 to $49,999, $50,000 to $74,999, and $75,000 and above. Respondents were asked if they had a personal physician; those with one or more were coded as yes. Health status was dichotomized into 1) fair or poor or 2) good, very good, or excellent.

### Statistical analysis

Stata, Version 8.0 (StataCorp, College Station, Tex), was used in all statistical analyses to account for the complex sample design of the BRFSS. Most analyses were performed on subpopulations representing four age and sex groups: men aged 50 to 64, women aged 50 to 64, men aged 65 and older, and women aged 65 and older. Pearson's chi-square tests were used to compare the percentage of adults who were up-to-date on the recommended services for their age and sex group by demographic characteristics. For mapping purposes, we divided state results into quartiles and then combined the middle two quartiles.

## Results

The median state response rate for the 2002 BRFSS was 58.3% (range 42.2%–82.6%) (11). Results for the individual preventive health services for each of the age and sex groups are presented in Appendix Tables A–D. All other results are for being up-to-date on cancer screening and vaccinations as defined above.

We combined data for 49 states and the District of Columbia to examine the percentage of adults who were up-to-date among demographic and risk-factor subgroups (Table 2). Black, Hispanic, and Asian older adults were significantly less likely than whites to be up-to-date in at least two of the four age and sex groups. On the other hand, although they did not reach statistical significance, rates for American Indians were consistently close to rates for whites in three of the four groups.

Among both men and women in all age groups, having more education was strongly related to being up-to-date. Respondents with less than a high school education were much less likely to be up-to-date (range 12.4%–29.5%) than those with a college degree (range 25.4%–43.8%). Similarly, higher income was associated with being up-to-date on cancer screening and vaccinations. Adults with health insurance were about twice as likely to almost three times as likely to be up-to-date as adults with no insurance. The same was true for adults with a personal physician compared with those without one. Overall, people who reported fair or poor health were more likely to be up-to-date than those who reported good, very good, or excellent health.

**Figure 1:**
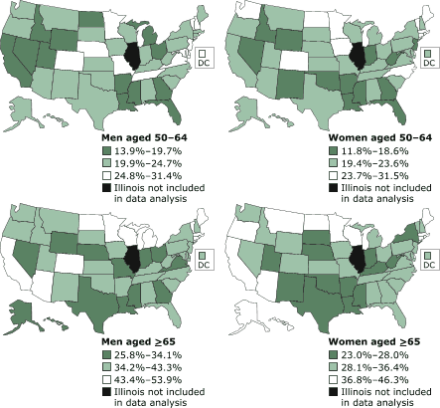
Prevalence estimates of adults aged ≥50 years who were up-to-date for cancer screening and vaccinations by age group, sex, and state, Behavioral Risk Factor Surveillance System (BRFSS), 2002. Data from 49 states and the District of Columbia. Results were divided into quartiles, and the middle two quartiles were combined. Illinois data are not included because the state used a split sample for some preventive service questions.

State-specific prevalence estimates for each of the age and sex groups were divided into quartiles and mapped (Figure 1). States in the western and southeastern United States were among those with the lowest percentage of men aged 50 to 64 who were up-to-date on cancer screening and vaccinations (California, Idaho, Nevada, Utah, and Wyoming in the West; Arkansas, Florida, Georgia, Louisiana, and Mississippi in the Southeast). There was a similar but less pronounced pattern for women aged 50 to 64 (Idaho, Nevada, and Wyoming in the West; Arkansas, Florida, Georgia, Louisiana, and Mississippi in the Southeast). The percentage of men and women aged 65 and older who were up-to-date was low in Indiana, Louisiana, Mississippi, Nebraska, and Texas. Arizona, California, Minnesota, North Dakota, Connecticut, Rhode Island, Wisconsin, Massachusetts, and Maine had higher percentages of people aged 65 and older who were up-to-date than most of the other states. Minnesota had the highest percentage of up-to-date adults in each of the four age and sex groups.

**Table:** 

**Women ≥65 years**	
**State**	**% Up-to-Date**
LA	23.0
IN	23.2
WV	24.4
OK	25.7
SD	26.5
NE	26.7
NM	26.7
KY	26.8
TX	27.1
AR	27.2
MS	27.4
UT	27.8
NY	28.0
GA	28.1
KS	28.5
MO	29.4
ID	29.5
OH	29.5
AL	29.8
WY	30.0
TN	30.3
DC	31.0
FL	32.4
VA	32.5
PA	32.6
MI	33.2
NJ	33.3
MD	33.6
NC	34.0
CO	34.0
HI	34.1
NV	34.2
NH	34.2
IA	34.3
VT	35.0
SC	36.1
WA	36.4
AK	36.8
MA	37.4
DE	37.4
ND	37.7
ME	37.8
RI	37.9
AZ	38.0
CA	38.1
WI	38.7
CT	39.0
OR	39.1
MT	39.7
MN	46.3

**Men 50-64 Years**	
**State**	**% Up-to-Date**
LA	13.9
GA	15.5
FL	15.6
NV	16.9
UT	17.2
OH	17.8
CA	18.0
ND	18.7
MI	19.0
AR	19.3
WY	19.3
MS	19.7
ID	19.7
SC	19.9
NM	20.0
AL	20.1
OR	20.2
KS	20.6
TX	20.6
HI	20.7
MT	21.1
WV	21.7
KY	22.3
PA	22.6
AK	22.7
NC	22.9
OK	23.1
NY	23.2
MA	23.2
WA	23.5
SD	23.7
IN	23.7
WI	23.8
ME	23.9
IA	24.2
AZ	24.4
CT	24.7
NJ	24.8
RI	25.1
MO	25.3
NE	25.4
VT	25.6
VA	25.9
MD	26.1
NH	26.2
CO	26.7
DC	27.8
TN	28.7
DE	30.4
MN	31.4

**Women 50-64 Years**	
**State**	**% Up-to-Date**
NV	11.8
LA	14.7
NJ	15.2
MS	15.8
FL	15.9
WY	16.3
AR	16.4
NM	16.8
WV	17.1
IN	17.3
ID	17.6
GA	18.3
OK	18.6
TX	19.4
PA	19.5
KY	20.0
OH	20.2
CA	21.0
MT	21.1
HI	21.2
MO	21.3
OR	21.3
DC	21.4
VA	21.4
AL	22.0
AK	22.2
MI	22.4
SC	22.6
SD	22.7
UT	22.8
AZ	22.9
WI	23.1
NH	23.3
TN	23.3
KS	23.4
NY	23.6
NE	23.6
MD	23.7
WA	24.5
VT	24.6
IA	24.9
MA	24.9
NC	25.3
RI	25.7
ND	25.9
DE	27.7
CO	27.9
CT	28.6
ME	29.7
MN	31.5

**Figure 2:**
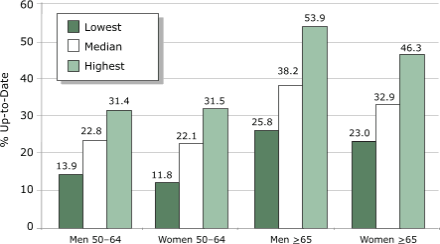
Median and range of state-specific results for percentage of adults aged ≥50 years who were up-to-date for cancer screening and immunizations, Behavioral Risk Factor Surveillance System (BRFSS), 2002. Data from 49 states and the District of Columbia. Illinois data are not included because the state used a split sample for some preventive service questions.

The percentage of each age and sex group that was up-to-date is presented by state in Table 3. The state medians for the percentage of respondents who were up-to-date were consistently less than 40% (with a range of 22.1% for women aged 50 to 64 to 38.2% for men aged 65 and older) (Figure 2). Median state values were similar to results for all adults reported in Table 2.

**Table:** 

Table 4 shows the percentage of adults who had zero, one, two, three, four, or five cancer screenings or vaccinations. Most adults had one or more preventive services, with the percentage of adults who received none of the services ranging from 3.6% for women aged 65 and older to 38.3% for men aged 50 to 64. Thus, the results indicated that at least 61.7% of older men and more than 95% of older women have had some contact with the health care system within the past few years.

## Discussion

This analysis of state BRFSS data indicates that among the four age and sex groups, the state medians for adults who are up-to-date with recommended cancer screenings and vaccinations range from 22.1% (women aged 50 to 64) to 38.2% (men aged 65 and older). Although the delivery rates of individual clinical preventive services may be relatively high — cervical cancer screening, for example, is at a national median of 81.0% — overall levels of protection provided by cancer screenings and vaccinations are low.

This study did not include data on cholesterol screening or tetanus–diphtheria vaccination, or data on clinical preventive services that are also recommended according to a specific schedule. Other excluded measures included screening for abnormalities of blood pressure, height, weight, obesity, vision, and hearing; chemoprevention for cardiovascular disease (aspirin); and counseling on calcium intake, folic acid, tobacco cessation, drug and alcohol use, sexually transmitted diseases, human immunodeficiency virus (HIV), nutrition, physical activity, sun exposure, oral health, injury prevention, and polypharmacy.

In addition to low absolute levels, the data indicate important differences among states. For each of the four age and sex groups, Minnesota consistently had the highest up-to-date percentages. Several factors may explain why this state has done so well in preventive service delivery: a high proportion of the population is enrolled in managed care; the state has a low uninsurance rate; and state-sponsored outreach promotes colorectal cancer screening and adult vaccinations. Additional research is necessary to fully explore the reasons for success in Minnesota. Many of the lowest measurements were found in the southeastern and south-central United States; these states have low rates of health insurance among adults (12).

Each of the four age and sex groups has its own set of recommended clinical preventive services. Figure 2 illustrates that men and women within each of the two age groups have similar proportions that are up-to-date, despite the fact that more tests are required of women. When compared with women in the same age group, men are much more likely to have had none of the recommended services. The reasons for these differences are not clear from the data and require further analysis.

There were significant differences in being up-to-date among racial and ethnic categories. Based on consistency across age and sex groups, it appears that blacks, Hispanics, and Asians may be less likely than whites to be up-to-date for cancer screenings and adult vaccinations. There may be higher rates of screening among American Indians because many American Indians receive health care through the Indian Health Service, which emphasizes preventive health practices. Further investigation of these racial and ethnic disparities is warranted.

Not only are reasons for racial and ethnic disparities not clear from the data but these disparities also are confounded by disparities in being up-to-date according to educational, socioeconomic, and insurance status. Respondents who have a college degree are 1.5 to 2 times more likely to be up-to-date than respondents who do not have a high school degree. Receipt of clinical preventive services is also consistently associated with higher income level for each of the four age and sex groups. Large differences exist between people who have medical insurance and people who do not. People with health insurance or personal physicians have approximately two to three times the rate of clinical preventive service delivery as people without either of them. According to the U.S. Census, 11.1% of whites, 19.6% of blacks, 18.8% of Asians, and 32.7% of Hispanics lacked insurance in 2003 (13). These findings are consistent with those of other studies that have found significant disparities in the delivery of cancer screening (14-16) and adult vaccinations (17,18) when measured by race/ethnic group, education, and income.

More than 95% of adults aged 65 and older have health insurance through Medicare, which pays for cancer screening and vaccinations (19). Nonetheless, fewer than 40% of this age group are up-to-date on all of the recommended cancer screening and vaccinations. Having health insurance (and a personal physician) — although necessary — is not in itself sufficient for achieving high levels of being up-to-date with cancer screenings and vaccinations.

Our results suggest that most respondents have received at least one cancer screening or vaccination. Therefore, most adults aged 50 and older have had some contact with the health care system within the past few years, but they do not receive the preventive services recommended for their age and sex group. At the same time, the analysis indicates that up-to-date rates are not low because adults lack only a single service.

This work builds on the analysis of others (20). The BRFSS has followed state rates for clinical preventive service delivery for more than 20 years, and a recent study has provided a comprehensive overview of trends (21). Combined measurements of mammography, clinical breast exam, and Pap testing have been made using data from the 1990 NHIS of Health Promotion and Disease Prevention (22). A methodology for building a prevention index has also been developed using electronic medical records. This index can serve as a combined quality assessment measure and be compared with consensus measures or with selected Health Plan Employer Data and Information Set (HEDIS) scores for health management organizations (23).

This study has a number of limitations. First, the BRFSS relies on self-reported data. Depending on the measure, self-reports can result in overestimates or underestimates compared with other data sources, such as health care records. Unfamiliarity with medical terms and telescoping (24) (recalling events more recently than they actually occurred) can affect the validity of self-reported clinical preventive services and probably result in overestimates (20,25). A review article by Nelson et al rates the validity of self-reports for mammography, Pap test, colorectal cancer screening, and pneumococcal vaccination as moderate and the validity for influenza vaccination as high (26). Second, households without telephones, which are more likely to have adults with low socioeconomic status, were excluded from the survey. This omission may have resulted in an overestimation of delivery rates (27). Third, the survey excludes people who rely only on cellular telephones for their telephone service. Finally, the BRFSS questions limited our ability to adequately determine compliance with Pap test recommendations for women aged 65 and older. The USPSTF recommends against routine screening for cervical cancer for women in this age group if they have had sufficient recent Pap tests with no abnormal results and are not otherwise at increased risk for cervical cancer (28). Without Pap test histories, we could not ascertain which of these older women might not need further Pap testing, and thus we were unable to determine how this anomaly in the recommendations might have affected our estimates of being up-to-date on the Pap test among women aged 65 and older. 

The results of this study suggest several potential next steps. From a surveillance perspective, we recommend that state-based surveys such as the BRFSS consider periodically collecting in the same year information about clinical preventive services with specific schedules (i.e., the services analyzed in this study as well as cholesterol screening and tetanus–diphtheria vaccination). In this way, a more complete measure of delivered adult clinical preventive services recommended by age and sex can be calculated. One of the strengths of a composite measure of clinical preventive services is that it emphasizes the overall protection of individuals by combining measurements across disease categories. We also recommend that surveys such as the BRFSS examine the feasibility and implications of routinely reporting a composite measure such as the one suggested by this study. 

The building blocks of composite measures will change as recommendations for individual interventions evolve. For example, colorectal cancer screening guidelines for *Healthy People 2010* objectives have not yet been updated to match USPSTF recommendations. (*Healthy People 2010* objectives for Pap test, mammography, and adult vaccinations are consistent with USPSTF recommendations for women aged 50 and older.)

The results of this study also suggest that the delivery of clinical preventive services in physicians' offices needs continued attention. Among respondents who indicated having a personal physician, the group with the highest proportion of up-to-date was men aged 65 and older at only 41.5%; the group with the lowest proportion was women aged 50 to 64 at 22.8%. Despite important initiatives designed to improve the provision of clinical preventive services in physicians' offices, such as the Agency for Healthcare Research and Quality's Put Prevention Into Practice (PPIP) (29), more work is necessary to enhance chart-flagging systems and improve reminder protocols aimed at patients. Physicians have begun to systematically consider various approaches to addressing multiple behavioral risk factors in primary care (30), and we hope that this commitment can be extended to providing multiple clinical preventive services.

From a public health standpoint, new kinds of programs are needed to address the shortfall identified by this study. Access to each of the recommended clinical preventive services can be facilitated inside and outside of the clinical setting. New initiatives must focus on populations, not patient panels. A community-wide strategy has been developed by the Sickness Prevention Achieved through Regional Collaboration (SPARC) program in Connecticut, Massachusetts, and New York, and has achieved promising results (31,32) by expanding delivery at clinical sites and creating new points of access in nonclinical settings. A similar approach has been taken by New York State's Healthy Women Partnerships, which facilitates the delivery of multiple cancer screenings for underserved populations. Community-wide assurance of clinical preventive services has been a priority for the Group Health Cooperative, a nonprofit health care system based in Seattle, Wash, which covers a large proportion of its region's residents (33).

Additional analytic work is necessary. An examination of data from states that collect information on cholesterol screening and tetanus–diphtheria vaccinations will yield a more complete — and perhaps lower — composite measure of up-to-date use of recommended services. A multivariable regression analysis will provide a fuller understanding of factors that are most strongly and independently associated with failure to be up-to-date, and a longitudinal analysis of trends in the composite measure will yield useful information for targeting state preventive health efforts.

This study reports low composite rates of adult clinical preventive service delivery based on 2002 BRFSS data for adults aged 50 and older and highlights significant disparities among groups. We must redouble our efforts to develop more effective approaches to delivering these basic adult clinical preventive services.

## Figures and Tables

**Table 1:** Clinical Preventive Services Recommended for Adults Aged 50 Years and Older by the United States Preventive Services Task Force (USPSTF), 2004^a^

**Measure**	**Definition**	**Age Groups for Which Services Are Recommended**	**Men 50-64**	**Women 50-64**	**Men ≥65**	**Women ≥65**
Influenza vaccination	Received in past 12 months	Men and women ≥50	X	X	X	X
Pneumococcal vaccination	Ever received	Men and women ≥65			X	X
Mammogram	Received in past 2 years	Women ≥40		X		X
Pap test	Received in past 3 years	Women ≥18 with intact cervix		X		X
Colorectal cancer screening^b^	Received FOBT in past 12 months or endoscopy in past 10 years	Men and women ≥50	X	X	X	X
Cholesterol screening^c^	Every 5 years	Men ≥35; women ≥45	X	X	X	X
Tetanus– diphtheria vaccination^c^	Every 10 years	Men and women ≥18 years	X	X	X	X

a Empty cells indicate that the service does not apply to the demographic category.

b Includes receiving fecal occult blood test (FOBT) or endoscopy (sigmoidoscopy or colonoscopy) within recommended time frames.

c These services are recommended by the USPSTF, but data were not collected by all states in the Behavioral Risk Factor Surveillance System (BRFSS), 2002, and were not analyzed as part of this study.

**Table 2:** Respondents Aged 50 Years and Older Who Are Up-To-Date for Cancer Screening and Adult Immunization by Age and Sex Group and Demographic Characteristics, Behavioral Risk Factor Surveillance System (BRFSS), 2002^a^

	**Men 50-64^b^** **% (95% CI)**	**Women 50-64^c^** **% (95% CI)**	**Men ≥65^d^** **% (95% CI)**	**Women ≥65^e^** **% (95% CI)**	**All Adults ≥50** **% (95% CI)**

**Race/ethnicity**					

White (non-Hispanic)	22.7 (21.8-23.7)	22.2 (21.4-23.0)	41.7 (40.3-43.0)	34.6 (33.6-35.6)	29.2 (28.7-29.7)
Black (non-Hispanic)	18.6 (15.2-22.6)	16.5 (14.1-19.3)	26.2 (20.9-32.3)	21.0 (17.4-25.2)	19.4 (17.7-21.3)
Hispanic (all races)	13.3 (9.8-17.8)	17.9 (14.3-22.1)	30.8 (22.0-41.2)	22.2 (15.2-31.3)	19.1 (16.4-22.2)
Asian or Pacific Islander^f^	12.4 (7.8-19.0)	20.1 (12.2-31.3)	25.7 (17.4-36.3)	21.1 (10.6-37.7)	17.6 (13.3-22.9)
American Indian or Alaska Native^f^	25.4 (14.4-40.9)	17.7 (10.3-28.9)	41.0 (25.0-59.1)	28.2 (12.1-52.8)	26.6 (19.8-34.6)
*P *value^g^	<.001	.03	<.001	<.001	<.001

**Education**					

<High school degree	12.4 (10.1-15.0)	15.5 (13.2-18.1)	29.5 (26.6-32.6)	22.7 (20.0-25.5)	20.4 (19.0-21.8)
High school degree or general equivalency diploma (GED)	18.8 (17.3-20.4)	18.5 (17.4-19.8)	38.5 (36.0-41.0)	30.5 (29.1-31.9)	25.5 (24.7-26.3)
Some college	22.0 (20.2-24.0)	21.9 (20.5-23.4)	43.8 (40.9-46.9)	38.4 (36.3-40.5)	29.4 (28.3-30.4)
College degree	25.4 (23.8-27.0)	25.4 (23.9-27.1)	43.8 (41.5-46.1)	38.9 (36.3-41.5)	30.9 (29.9-31.8)
*P* value^g^	<.001	<.001	<.001	<.001	<.001

**Annual income,$^h^**					

<25,000	16.9 (15.0-19.1)	16.4 (15.0-17.8)	35.1 (32.7-37.5)	27.2 (25.8-28.7)	24.2 (23.3-25.1)
25,000-49,999	19.4 (17.8-21.1)	21.1 (19.7-22.6)	42.4 (40.0-44.7)	37.2 (35.1-39.4)	28.9 (27.9-29.8)
50,000-74,999	21.7 (19.7-23.8)	22.0 (20.2-23.9)	43.1 (39.2-47.2)	40.5 (35.3-45.9)	26.9 (25.6-28.3)
≥75,000	25.6 (23.8-27.5)	25.3 (23.4-27.4)	42.4 (38.4-46.4)	43.5 (38.1-49.1)	28.6 (27.4-30.0)
*P *value^g^	<.001	<.001	<.001	<.001	<.001

**Insured**					

Yes	23.3 (22.3-24.3)	23.1 (22.3-24.0)	40.1 (38.7-41.4)	32.8 (31.8-33.8)	28.9 (28.4-29.4)
No	7.9 (6.6-9.5)	7.8 (6.4-9.5)	19.1 (13.9-25.6)	16.5 (11.5-23.3)	9.0 (8.0-10.2)
*P* value	<.001	<.001	<.001	<.001	<.001

**Personal physician**					

Yes	24.0 (22.9-25.0)	22.8 (21.9-23.6)	41.5 (40.1-42.9)	33.8 (32.8-34.9)	29.3 (28.8-29.8)
No	8.0 (6.7-9.6)	6.6 (5.0-8.5)	20.7 (16.8-25.2)	12.2 (9.7-15.1)	10.1 (9.1-11.3)
*P* value	<.001	<.001	<.001	<.001	<.001

**Health status**					

Fair or poor	26.2 (24.0-28.6)	22.0 (20.3-23.8)	41.5 (39.0-44.1)	31.6 (29.6-33.6)	30.0 (28.9-31.1)
Good, very good, or excellent	20.4 (19.4-21.4)	20.9 (20.0-21.8)	38.9 (37.3-40.4)	32.9 (31.7-34.1)	26.5 (25.9-27.0)
*P* value	<.001	.27	.08	.29	.001

**Total**	21.4 (20.5-22.3)	21.1 (20.3-21.9)	39.6 (38.2-40.9)	32.4 (31.4-33.4)	27.3 (26.8-27.8)

No. BRFSS respondents aged ≥50 (%)	23,568 (22.3)	33,389 (31.5)	17,187 (16.2)	31,716 (30.0)	105,860
No. BRFSS respondents aged ≥50 with missing data other than annual income (%)^h^	609 (2.6)	982 (2.9)	1,327 (7.7)	2,779 (8.8)	5,697 (5.4)
No. BRFSS respondents aged ≥50 with complete data (except annual income)^h^	22,959	32,407	15,860	28,937	100,163

a N = 100,163; includes complete sets of data from 49 states and the District of Columbia. Illinois data are not included because the state used a split sample for some preventive services questions. CI indicates confidence interval.

b Men aged 50–64 were up-to-date for preventive services if they met screening recommendations for colorectal cancer and had an influenza vaccination in the past year.

c Women aged 50–64 were up-to-date for preventive services if they met screening recommendations for breast cancer, cervical cancer, colorectal cancer, and had an influenza vaccination in the past year.

d dMen aged ≥65 were up-to-date for preventive services if they met screening recommendations for colorectal cancer, had an influenza vaccination in the past year, and had ever had a pneumococcal vaccination.

e Women aged ≥65 were up-to-date on preventive services if they met screening recommendations for breast cancer, cervical cancer, and colorectal cancer, had an influenza vaccination in the past year, and had ever had a pneumococcal vaccination.

f These estimates are based on small numbers of respondents, resulting in wide confidence intervals and potentially unstable point estimates, and should be interpreted with caution.

g *P* values <.05 indicate that being up-to-date is associated with a demographic characteristic within an age and sex group based on Pearson's chi-square test but do not specify which groups are significantly different from each other.

h N = 82,769 for annual income; 17.4% (17,394/100,163) of otherwise complete questionnaires were missing data on annual income.

**Table 3:** Percentage of Respondents Who Are Up-To-Date for Cancer Screening and Vaccinations, by State, Behavioral Risk Factor Surveillance System (BRFSS), 2002

	**Men 50-64^a^**		**Women 50-64^b^**		**Men ≥65^c^**		**Women ≥65^d^**	

	**N**	**% (95% CI)^e^**	**N**	**% (95% CI)**	**N**	**% (95% CI)**	**N**	**% (95% CI)**
Alabama	265	20.1 (15.3-25.9)	486	22.0 (18.4-26.2)	206	34.7 (27.9-42.2)	482	29.8 (25.4-34.7)
Alaska	299	22.7 (16.3-30.6)	323	22.2 (15.7-30.3)	127	25.8 (16.3-38.4)	166	36.8 (25.6-49.7)
Arizona	326	24.4 (17.9-32.2)	512	22.9 (18.3-28.3)	331	48.8 (41.3-56.3)	527	38.0 (32.0-44.4)
Arkansas	380	19.3 (15.4-24.0)	598	16.4 (13.5-19.8)	321	38.2 (32.5-44.2)	600	27.2 (23.3-31.4)
California	385	18.0 (14.2-22.5)	531	21.0 (17.3-25.3)	277	45.7 (38.6-53.0)	472	38.1 (32.3-44.2)
Colorado	371	26.7 (21.2-33.1)	602	27.9 (24.1-32.0)	234	48.3 (41.2-55.4)	432	34.0 (28.7-39.7)
Connecticut	592	24.7 (20.6-29.3)	712	28.6 (24.6-32.9)	392	44.3 (38.5-50.3)	708	39.0 (34.7-43.5)
Delaware	373	30.4 (24.2-37.4)	552	27.7 (23.2-32.7)	348	38.8 (32.3-45.7)	575	37.4 (32.3-42.9)
District of Columbia	210	27.8 (21.1-35.6)	282	21.4 (16.4-27.4)	146	34.5 (25.8-44.4)	259	31.0 (24.6-38.3)
Florida	574	15.6 (12.7-19.0)	869	15.9 (13.3-18.8)	600	36.6 (32.4-41.1)	957	32.4 (29.0-36.0)
Georgia	430	15.5 (11.9-19.9)	714	18.3 (15.1-22.0)	290	33.0 (27.1-39.5)	598	28.1 (23.8-32.8)
Hawaii	661	20.7 (16.6-25.6)	798	21.2 (17.6-25.3)	485	33.8 (28.5-39.6)	764	34.1 (29.6-38.8)
Idaho	505	19.7 (15.8-24.3)	646	17.6 (14.4-21.2)	388	34.2 (28.9-39.8)	688	29.5 (25.4-34.0)
Illinois^f^	---	---	---	---	---	---	---	---
Indiana	508	23.7 (19.8-28.1)	830	17.3 (14.7-20.3)	388	33.3 (28.3-38.8)	710	23.2 (19.8-27.0)
Iowa	337	24.2 (19.4-29.8)	517	24.9 (20.8-29.4)	291	42.0 (35.7-48.6)	630	34.3 (29.8-39.1)
Kansas	455	20.6 (16.8-25.0)	612	23.4 (19.9-27.4)	307	43.2 (37.0-49.5)	586	28.5 (24.3-33.0)
Kentucky	605	22.3 (17.7-27.8)	1097	20.0 (16.6-23.8)	506	36.3 (30.6-42.4)	1315	26.8 (23.2-30.8)
Louisiana	419	13.9 (10.3-18.3)	744	14.7 (12.2-17.6)	334	31.0 (25.7-36.9)	692	23.0 (19.7-26.7)
Maine	251	23.9 (18.6-30.1)	362	29.7 (24.7-35.1)	161	50.1 (41.5-58.6)	318	37.8 (31.9-44.0)
Maryland	424	26.1 (21.6-31.1)	635	23.7 (19.9-27.8)	289	46.4 (38.8-54.2)	478	33.6 (28.2-39.4)
Massachusetts	662	23.2 (19.6-27.3)	883	24.9 (21.6-28.4)	499	43.8 (38.5-49.2)	908	37.4 (33.3-41.6)
Michigan	607	19.0 (15.6-22.9)	855	22.4 (19.2-25.9)	435	43.4 (37.8-49.1)	740	33.2 (29.0-37.6)
Minnesota	479	31.4 (27.0-36.0)	590	31.5 (27.5-35.9)	305	53.9 (47.7-60.0)	640	46.3 (41.9-50.8)
Mississippi	373	19.7 (15.5-24.8)	607	15.8 (13.0-19.0)	248	28.9 (23.1-35.5)	647	27.4 (23.5-31.7)
Missouri	445	25.3 (20.0-31.6)	685	21.3 (17.3-25.8)	381	31.1 (25.4-37.5)	761	29.4 (24.9-34.3)
Montana	486	21.1 (15.9-27.4)	556	21.1 (17.0-25.7)	311	35.7 (28.9-43.2)	537	39.7 (34.1-45.6)
Nebraska	356	25.4 (20.8-30.7)	539	23.6 (19.8-27.9)	393	32.4 (27.4-37.7)	754	26.7 (23.0-30.7)
Nevada	400	16.9 (12.1-23.0)	366	11.8 (8.6-16.1)	266	32.9 (25.3-41.5)	337	34.2 (27.5-41.6)
New Hampshire	538	26.2 (22.1-30.6)	692	23.3 (20.0-26.9)	331	39.8 (34.0-45.8)	562	34.2 (29.9-38.9)
New Jersey	612	24.8 (18.1-32.9)	813	15.2 (10.5-21.5)	491	39.9 (31.6-48.9)	813	33.3 (26.8-40.4)
New Mexico	502	20.0 (16.3-24.3)	709	16.8 (13.8-20.2)	410	38.6 (33.2-44.3)	625	26.7 (22.4-31.6)
New York	399	23.2 (18.7-28.3)	575	23.6 (19.8-27.8)	289	43.3 (36.7-50.2)	532	28.0 (23.8-32.5)
North Carolina	618	22.9 (18.3-28.3)	948	25.3 (21.5-29.6)	485	40.4 (33.4-47.7)	944	34.0 (29.5-38.7)
North Dakota	325	18.7 (14.6-23.6)	375	25.9 (21.4-31.0)	206	46.5 (39.1-54.2)	439	37.7 (32.3-43.4)
Ohio	398	17.8 (13.9-22.4)	548	20.2 (16.7-24.3)	262	34.1 (27.6-41.3)	513	29.5 (24.7-34.9)
Oklahoma	709	23.1 (19.5-27.0)	1024	18.6 (16.2-21.3)	605	38.2 (34.0-42.6)	1083	25.7 (22.8-28.8)
Oregon	328	20.2 (15.8-25.3)	467	21.3 (17.6-25.6)	213	34.5 (27.7-41.9)	408	39.1 (33.8-44.7)
Pennsylvania	1351	22.6 (19.9-25.6)	1818	19.5 (17.3-22.0)	999	40.2(36.4-44.2)	1985	32.6 (29.9-35.5)
Rhode Island	330	25.1 (20.3-30.5)	500	25.7 (21.7-30.1)	273	48.4 (41.9-55.0)	477	37.9 (33.1-43.1)
South Carolina	460	19.9 (15.8-24.7)	666	22.6 (18.7-27.0)	319	42.6 (35.8-49.7)	583	36.1 (31.0-41.5)
South Dakota	462	23.7 (19.8-27.9)	634	22.7 (19.2-26.7)	415	35.2 (30.1-40.6)	775	26.5 (23.3-30.1)
Tennessee	302	28.7 (23.4-34.6)	462	23.3 (19.4-27.7)	249	38.1 (31.5-45.2)	423	30.3 (25.5-35.6)
Texas	489	20.6 (16.9-24.8)	772	19.4 (16.5-22.6)	359	31.9 (26.7-37.5)	672	27.1 (23.2-31.4)
Utah	409	17.2 (13.1-22.3)	449	22.8 (18.0-28.5)	285	41.9 (35.1-48.9)	403	27.8 (22.2-34.1)
Vermont	477	25.6 (21.6-30.1)	570	24.6 (21.0-28.6)	312	49.9 (43.8-56.1)	509	35.0 (30.4-39.9)
Virginia	422	25.9 (20.3-32.3)	606	21.4 (17.3-26.1)	292	33.9 (26.8-41.9)	513	32.5 (27.7-37.8)
Washington	478	23.5 (19.2-28.4)	679	24.5 (20.8-28.6)	324	36.5 (30.7-42.7)	597	36.4 (31.6-41.6)
West Virginia	336	21.7 (17.4-26.8)	504	17.1 (13.8-21.0)	276	35.7 (29.7-42.1)	570	24.4 (20.6-28.6)
Wisconsin	427	23.8 (19.5-28.7)	576	23.1 (19.4-27.3)	282	45.4 (38.6-52.4)	565	38.7 (33.9-43.6)
Wyoming	409	19.3 (15.5-23.9)	517	16.3 (13.2-20.0)	251	33.1 (27.0-39.8)	444	30.0 (25.5-34.9)

Lowest		13.9		11.8		25.8		23.0
Highest		31.4		31.5		53.9		46.3
Median^g^		22.8		22.1		38.2		32.9

a Men aged 50–64 were up-to-date for preventive services if they met screening recommendations for colorectal cancer and had an influenza vaccination in the past year.

b Women aged 50–64 were up-to-date for preventive services if they met screening recommendations for breast cancer, cervical cancer, colorectal cancer, and had an influenza vaccination in the past year.

c Men aged ≥65 were up-to-date for preventive services if they met screening recommendations for colorectal cancer, had an influenza vaccination in the past year, and had ever had a pneumococcal vaccination.

d Women aged ≥65 were up-to-date for preventive services if they met screening recommendations for breast cancer, cervical cancer, and colorectal cancer, had an influenza vaccination in the past year, and had ever had a pneumococcal vaccination.

e CI indicates confidence interval.

f Illinois data are not included because the state used a split sample for some preventive services questions.

g Median represents the median value among the 49 states and the District of Columbia.

**Table 4:** Number of Preventive Services Received by Age and Sex Group, Behavioral Risk Factor Surveillance System (BRFSS), 2002^a^

	**No. Preventive Services Received**					

	**0**	**1**	**2**	**3**	**4**	**5**
Men aged 50-64^b^	38.3	40.3	21.4	NA^c^	NA	NA
Men aged ≥65^d^	12.6	20.5	27.3	39.6	NA	NA
Women aged 50-64^e^	4.7	9.0	28.2	37.0	21.1	NA
Women aged ≥65^f^	3.6	5.5	11.9	19.5	27.1	32.4

a All values represent percentages. Includes data from 49 states and the District of Columbia. Illinois data are not included because the state used a split sample for some preventive services questions.

b Men aged 50–64 were up-to-date for preventive services if they received two services (described in Table 1).

c NA indicates not applicable.

d Women aged 50–64 were up-to-date for preventive services if they received four services (described in Table 1).

e Women aged 50–64 were up-to-date for preventive services if they received four services (described in Table 1).

f Women aged ≥65 were up-to-date for preventive services if they received five services (described in Table 1).

## Appendices

### Appendix Table A. Prevalence of Receipt of Preventive Services for Men Aged 50–64 Years, Behavioral Risk Factor Surveillance System (BRFSS), 2002

**Table:**

	** **	**Colon Cancer Screening^a^**		**Influenza vaccination^b^**		**Up-to-Date^c^**	

	**N**	**%**	**(95% CI)^d^**	**%**	**(95% CI)**	**%**	**(95% CI)**
Alabama	273	46.5	(40.0-53.1)	35.3	(29.4-41.7)	20.1	(15.3-25.9)
Alaska	308	49.9	(41.5-58.3)	37.0	(29.4-45.3)	22.7	(16.3-30.6)
Arizona	330	49.0	(40.0-58.1)	31.8	(24.6-39.9)	24.4	(17.9-32.2)
Arkansas	390	38.1	(33.0-43.5)	37.3	(32.2-42.6)	19.3	(15.4-24.0)
California	393	43.7	(37.9-49.6)	31.1	(26.1-36.6)	18.0	(14.2-22.5)
Colorado	384	48.1	(42.0-54.2)	44.2	(38.3-50.3)	26.7	(21.2-33.1)
Connecticut	617	56.1	(51.0-61.1)	35.6	(31.0-40.4)	24.7	(20.6-29.3)
Delaware	376	58.2	(51.2-64.9)	45.7	(38.8-52.7)	30.4	(24.2-37.4)
District of Columbia	215	59.8	(51.5-67.7)	34.4	(27.3-42.4)	27.8	(21.1-35.6)
Florida	592	47.9	(43.0-52.7)	26.5	(22.6-30.7)	15.6	(12.7-19.0)
Georgia	445	45.1	(39.6-50.7)	30.8	(25.9-36.2)	15.5	(11.9-19.9)
Hawaii	675	41.4	(36.2-46.7)	34.4	(29.3-39.9)	20.7	(16.6-25.6)
Idaho	519	44.7	(39.8-49.8)	33.3	(28.7-38.3)	19.7	(15.8-24.3)
Illinois^e^	---	---	----	---	---	----	----
Indiana	518	45.2	(40.4-50.0)	40.8	(36.3-45.6)	23.7	(19.8-28.1)
Iowa	340	45.0	(39.1-51.0)	44.8	(39.0-50.7)	24.2	(19.4-29.8)
Kansas	466	48.8	(43.8-53.7)	35.2	(30.6-40.1)	20.6	(16.8-25.0)
Kentucky	642	46.0	(40.4-51.6)	38.5	(33.2-44.1)	22.3	(17.7-27.8)
Louisiana	437	42.6	(37.2-48.2)	26.6	(22.1-31.6)	13.9	(10.3-18.3)
Maine	257	53.1	(46.2-59.8)	36.7	(30.5-43.4)	23.9	(18.6-30.1)
Maryland	437	56.9	(51.0-62.5)	38.4	(33.1-43.9)	26.1	(21.6-31.1)
Massachusetts	693	59.2	(54.7-63.6)	35.8	(31.6-40.2)	23.2	(19.6-27.3)
Michigan	616	52.0	(47.1-56.8)	29.1	(25.1-33.5)	19.0	(15.6-22.9)
Minnesota	482	60.7	(55.9-65.2)	42.7	(38.0-47.5)	31.4	(27.0-36.0)
Mississippi	394	39.7	(34.4-45.3)	36.3	(31.2-41.7)	19.7	(15.5-24.8)
Missouri	451	52.5	(46.1-58.8)	38.8	(32.7-45.3)	25.3	(20.0-31.6)
Montana	502	38.9	(32.9-45.2)	40.7	(34.8-46.9)	21.1	(15.9-27.4)
Nebraska	365	46.7	(41.0-52.4)	40.7	(35.3-46.4)	25.4	(20.8-30.7)
Nevada	410	41.7	(34.6-49.1)	27.1	(21.3-33.8)	16.9	(12.1-23.0)
New Hampshire	553	56.5	(51.8-61.1)	37.5	(33.0-42.1)	26.2	(22.1-30.6)
New Jersey	631	50.4	(42.3-58.6)	38.0	(30.4-46.3)	24.8	(18.1-32.9)
New Mexico	513	42.7	(37.8-47.8)	37.8	(33.1-42.8)	20.0	(16.3-24.3)
New York	417	54.0	(48.0-59.9)	35.7	(30.3-41.5)	23.2	(18.7-28.3)
North Carolina	631	49.6	(43.9-55.3)	37.7	(32.3-43.3)	22.9	(18.3-28.3)
North Dakota	333	35.4	(30.2-41.1)	32.8	(27.7-38.3)	18.7	(14.6-23.6)
Ohio	414	47.6	(41.8-53.5)	29.3	(24.5-34.7)	17.8	(13.9-22.4)
Oklahoma	718	36.2	(32.3-40.4)	46.5	(42.3-50.7)	23.1	(19.5-27.0)
Oregon	336	47.3	(41.3-53.3)	35.4	(29.9-41.3)	20.2	(15.8-25.3)
Pennsylvania	1388	47.1	(43.7-50.5)	37.3	(34.1-40.6)	22.6	(19.9-25.6)
Rhode Island	335	53.8	(47.7-59.8)	39.4	(33.7-45.3)	25.1	(20.3-30.5)
South Carolina	471	45.2	(39.4-51.2)	34.5	(29.2-40.2)	19.9	(15.8-24.7)
South Dakota	469	36.6	(32.0-41.5)	48.1	(43.0-53.2)	23.7	(19.8-27.9)
Tennessee	306	50.5	(44.0-57.0)	42.0	(35.9-48.4)	28.7	(23.4-34.6)
Texas	494	41.5	(36.7-46.5)	33.2	(28.4-38.5)	20.6	(16.9-24.8)
Utah	413	40.0	(34.0-46.4)	32.8	(27.3-38.9)	17.2	(13.1-22.3)
Vermont	488	58.0	(53.1-62.7)	35.2	(30.7-39.9)	25.6	(21.6-30.1)
Virginia	441	52.6	(45.8-59.4)	41.4	(34.7-48.4)	25.9	(20.3-32.3)
Washington	494	51.4	(45.7-57.1)	37.5	(32.4-43.0)	23.5	(19.2-28.4)
West Virginia	340	42.0	(36.4-47.7)	37.4	(32.0-43.1)	21.7	(17.4-26.8)
Wisconsin	441	55.1	(49.6-60.4)	36.0	(31.0-41.2)	23.8	(19.5-28.7)
Wyoming	415	34.5	(29.5-40.0)	41.1	(35.2-47.3)	19.3	(15.5-23.9)

Lowest		34.5		26.5		13.9	
Highest		60.7		48.1		31.4	
Median^f^		47.4		36.9		22.8	

a Met colorectal cancer screening recommendation (had either fecal occult blood testing [FOBT] in the past year or endoscopy [colonoscopy or sigmoidoscopy] within the past 10 years).

b Met immunization recommendation for influenza vaccination (influenza vaccination in the past year).

b Met recommendations for colorectal cancer screening and immunization.

d CI indicates confidence interval.

e Illinois data are not included because the state used a split sample for some preventive services questions.

f Median represents the median value among the 49 states and the District of Columbia.

### Appendix Table B. Prevalence of Receipt of Preventive Services for Women Aged 50–64 Years, Behavioral Risk Factor Surveillance System (BRFSS), 2002

**Table:**

	** **	**Breast Cancer Screening^a^**		**Cervical Cancer Screening^b^**		**Colon Cancer Screening^c^**		**Influenza Vaccination^d^**		**Up-to-Date^e^**	

	**N**	**%**	**(95% CI)^f^**	**%**	**(95% CI)**	**%**	**(95% CI)**	**%**	**(95% CI)**	**%**	**(95% CI)**
Alabama	499	80.1	(75.9-83.7)	87.1	(81.8-91.0)	48.3	(43.5-53.2)	39.1	(34.6-43.8)	22.0	(18.4-26.2)
Alaska	334	84.7	(77.6-89.8)	88.9	(78.9-94.5)	52.9	(43.4-62.1)	38.1	(29.7-47.3)	22.2	(15.7-30.3)
Arizona	516	81.4	(76.6-85.4)	87.2	(80.8-91.6)	47.3	(40.9-53.7)	41.0	(35.1-47.3)	22.9	(18.3-28.3)
Arkansas	624	69.7	(65.3-73.7)	75.3	(68.9-80.7)	38.0	(33.7-42.5)	40.5	(36.3-44.9)	16.4	(13.5-19.8)
California	542	83.0	(78.6-86.7)	85.4	(79.9-89.6)	47.7	(42.6-52.9)	36.7	(31.9-41.6)	21.0	(17.3-25.3)
Colorado	615	81.2	(77.4-84.4)	89.7	(85.9-92.6)	51.7	(47.2-56.1)	46.3	(41.9-50.7)	27.9	(24.1-32.0)
Connecticut	743	87.2	(83.6-90.1)	91.6	(88.4-94.0)	59.1	(54.5-63.5)	44.2	(39.8-48.8)	28.6	(24.6-32.9)
Delaware	586	90.7	(87.6-93.1)	94.3	(91.4-96.3)	57.7	(52.3-62.9)	42.5	(37.2-47.8)	27.7	(23.2-32.7)
District of Columbia	292	86.0	(80.7-90.0)	93.7	(88.5-96.7)	58.0	(50.9-64.7)	35.7	(29.3-42.5)	21.4	(16.4-27.4)
Florida	897	79.3	(75.7-82.5)	89.9	(86.1-92.7)	46.8	(42.8-50.7)	28.0	(24.6-31.6)	15.9	(13.3-18.8)
Georgia	737	80.7	(76.7-84.1)	89.7	(85.9-92.5)	51.4	(47.1-55.7)	34.3	(30.3-38.5)	18.3	(15.1-22.0)
Hawaii	819	74.4	(70.1-78.3)	83.2	(78.7-86.9)	42.9	(38.3-47.5)	38.9	(34.5-43.5)	21.2	(17.6-25.3)
Idaho	662	72.0	(68.0-75.7)	83.1	(78.1-87.1)	39.2	(34.9-43.6)	38.0	(33.9-42.3)	17.6	(14.4-21.2)
Illinois^g^	---	---	---	---	---	----	---	---	---	---	----
Indiana	851	79.7	(76.5-82.6)	85.0	(81.2-88.2)	40.6	(36.9-44.5)	40.5	(36.8-44.2)	17.3	(14.7-20.3)
Iowa	526	83.3	(79.3-86.6)	88.3	(83.6-91.7)	49.1	(44.2-53.9)	45.4	(40.6-50.3)	24.9	(20.8-29.4)
Kansas	623	81.6	(78.1-84.6)	88.0	(83.6-91.4)	44.6	(40.4-48.9)	47.3	(43.0-51.6)	23.4	(19.9-27.4)
Kentucky	1170	79.8	(76.4-82.8)	84.9	(80.9-88.2)	47.3	(43.0-51.6)	38.6	(34.6-42.8)	20.0	(16.6-23.8)
Louisiana	765	80.2	(76.9-83.1)	86.7	(82.2-90.2)	39.7	(35.9-43.6)	30.8	(27.4-34.5)	14.7	(12.2-17.6)
Maine	374	86.5	(82.3-89.8)	94.5	(90.8-96.8)	53.4	(47.7-58.9)	50.0	(44.4-55.5)	29.7	(24.7-35.1)
Maryland	654	88.2	(85.0-90.9)	91.9	(86.7-95.2)	55.6	(50.7-60.3)	41.1	(36.5-45.8)	23.7	(19.9-27.8)
Massachusetts	923	88.9	(86.1-91.1)	88.7	(85.3-91.3)	53.2	(49.1-57.3)	42.5	(38.5-46.5)	24.9	(21.6-28.4)
Michigan	870	82.9	(78.4-86.6)	88.3	(84.2-91.4)	54.1	(49.6-58.6)	35.0	(31.1-39.0)	22.4	(19.2-25.9)
Minnesota	599	86.3	(82.9-89.1)	91.4	(88.1-93.8)	58.8	(54.3-63.0)	45.2	(40.8-49.6)	31.5	(27.5-35.9)
Mississippi	637	73.1	(69.0-76.8)	83.8	(77.9-88.4)	38.6	(34.5-42.8)	34.7	(30.9-38.8)	15.8	(13.0-19.0)
Missouri	698	78.9	(74.7-82.6)	83.2	(77.7-87.6)	42.9	(38.0-47.9)	42.3	(37.5-47.3)	21.3	(17.3-25.8)
Montana	570	78.3	(74.0-82.0)	85.2	(80.3-89.0)	46.6	(41.2-52.0)	44.5	(39.3-49.8)	21.1	(17.0-25.7)
Nebraska	551	79.2	(75.1-82.7)	88.4	(84.3-91.5)	43.0	(38.5-47.6)	47.8	(43.3-52.4)	23.6	(19.8-27.9)
Nevada	378	78.7	(72.3-83.9)	89.8	(82.9-94.1)	40.8	(34.1-47.8)	31.2	(25.4-37.6)	11.8	(8.6-16.1)
New Hampshire	703	84.2	(80.9-87.0)	89.1	(85.6-91.9)	49.7	(45.6-53.8)	39.3	(35.4-43.4)	23.3	(20.0-26.9)
New Jersey	847	82.2	(76.6-86.7)	85.4	(78.9-90.1)	44.5	(37.4-51.9)	32.8	(26.5-39.7)	15.2	(10.5-21.5)
New Mexico	728	77.9	(74.2-81.2)	85.2	(81.0-88.6)	37.0	(33.0-41.2)	38.1	(34.1-42.3)	16.8	(13.8-20.2)
New York	600	86.2	(82.8-89.0)	91.0	(87.6-93.6)	50.7	(45.9-55.5)	39.1	(34.6-43.8)	23.6	(19.8-27.8)
North Carolina	978	85.3	(81.5-88.3)	91.8	(87.2-94.9)	57.4	(52.6-62.0)	41.4	(36.9-46.1)	25.3	(21.5-29.6)
North Dakota	387	81.8	(77.3-85.5)	85.4	(80.3-89.3)	45.5	(40.1-51.0)	47.1	(41.7-52.5)	25.9	(21.4-31.0)
Ohio	570	81.5	(77.7-84.8)	86.4	(81.7-90.0)	48.4	(43.7-53.1)	38.0	(33.6-42.7)	20.2	(16.7-24.3)
Oklahoma	1045	74.2	(71.1-77.1)	84.0	(80.3-87.2)	37.4	(34.1-40.7)	43.2	(39.8-46.6)	18.6	(16.2-21.3)
Oregon	478	79.8	(75.4-83.6)	86.9	(82.0-90.5)	45.6	(40.6-50.5)	39.7	(35.0-44.7)	21.3	(17.6-25.6)
Pennsylvania	1875	80.9	(78.4-83.2)	86.3	(83.5-88.6)	43.9	(40.9-46.9)	39.2	(36.4-42.1)	19.5	(17.3-22.0)
Rhode Island	515	90.7	(87.6-93.1)	92.6	(88.9-95.1)	55.3	(50.4-60.1)	43.3	(38.6-48.1)	25.7	(21.7-30.1)
South Carolina	688	79.0	(74.8-82.6)	85.3	(80.1-89.3)	47.5	(42.8-52.3)	39.9	(35.4-44.6)	22.6	(18.7-27.0)
South Dakota	642	83.2	(79.8-86.0)	89.9	(86.5-92.5)	42.2	(38.0-46.5)	49.8	(45.6-54.0)	22.7	(19.2-26.7)
Tennessee	474	78.6	(74.1-82.5)	83.1	(77.3-87.6)	49.3	(44.3-54.3)	43.9	(39.1-48.9)	23.3	(19.4-27.7)
Texas	799	77.9	(74.5-81.0)	85.2	(81.0-88.6)	42.6	(38.6-46.7)	42.0	(38.0-46.1)	19.4	(16.5-22.6)
Utah	456	79.0	(74.0-83.2)	85.6	(78.9-90.4)	41.5	(35.7-47.6)	47.1	(41.2-53.1)	22.8	(18.0-28.5)
Vermont	592	83.4	(79.7-86.6)	89.1	(85.5-91.9)	53.4	(49.0-57.8)	39.1	(35.0-43.4)	24.6	(21.0-28.6)
Virginia	633	78.2	(72.9-82.6)	87.1	(81.6-91.1)	45.1	(39.8-50.6)	38.4	(33.5-43.7)	21.4	(17.3-26.1)
Washington	694	80.4	(76.5-83.7)	91.0	(87.5-93.6)	55.4	(50.9-59.9)	40.1	(35.8-44.5)	24.5	(20.8-28.6)
West Virginia	512	81.6	(77.6-85.0)	86.6	(81.9-90.2)	36.6	(32.1-41.3)	40.0	(35.4-44.7)	17.1	(13.8-21.0)
Wisconsin	590	84.3	(80.6-87.4)	88.2	(84.1-91.4)	51.7	(46.9-56.3)	40.2	(35.7-44.8)	23.1	(19.4-27.3)
Wyoming	528	74.8	(70.6-78.6)	85.6	(80.9-89.4)	33.9	(29.7-38.4)	40.5	(36.1-45.1)	16.3	(13.2-20.0)

Lowest		69.7		75.3		33.9		28.0		11.8	
Highest		90.7		94.5		59.1		50.0		31.5	
Median^h^		81.0		87.1		47.3		40.1		22.1	

a Met breast cancer screening recommendations (mammogram in past 2 years).

b Met cervical cancer screening recommendations (Pap test in the past 3 years, unless the respondent had a hysterectomy and a Pap test was not needed).

c Met colorectal cancer screening recommendations (either fecal occult blood testing [FOBT] in the past year or endoscopy [colonoscopy or sigmoidoscopy] within the past 10 years).

d Met recommendation for influenza vaccination (influenza vaccination in the past year).

e Up-to-date on preventive services: met screening recommendations for breast cancer, cervical cancer (or if the respondent had a hysterectomy and cervical cancer screening was not recommended), colorectal cancer, and vaccination.

f CI indicates confidence interval.

g Illinois data are not included because the state used a split sample for some preventive services questions.

h Median represents the median value among the 49 states and the District of Columbia.

### Appendix Table C. Prevalence of Receipt of Preventive Services for Men Aged 65 Years and Older, Behavioral Risk Factor Surveillance System (BRFSS), 2002

**Table:**

	** **	**Colon Cancer Screening^a^**		**Influenza Vaccination^b^**		**Pneumococcal Vaccination^c^**		**Up-to-Date^d^**	

	**N**	**%**	**(95% CI)^e^**	**%**	**(95% CI)**	**%**	**(95% CI)**	**%**	**(95% CI)**
Alabama	206	59.6	(52.1-66.7)	64.6	(57.3-71.3)	57.5	(50.0-64.6)	34.7	(27.9-42.2)
Alaska	127	72.9	(61.4-82.0)	63.5	(49.3-75.7)	47.5	(33.9-61.5)	25.8	(16.3-38.4)
Arizona	331	77.5	(71.2-82.8)	72.2	(65.2-78.3)	64.8	(57.6-71.4)	48.8	(41.3-56.3)
Arkansas	321	57.1	(51.1-63.0)	70.2	(64.5-75.4)	58.2	(52.3-63.9)	38.2	(32.5-44.2)
California	277	73.0	(65.9-79.1)	69.8	(63.3-75.7)	59.9	(52.8-66.6)	45.7	(38.6-53.0)
Colorado	234	68.7	(61.9-74.9)	76.7	(70.2-82.2)	66.4	(59.5-72.8)	48.3	(41.2-55.4)
Connecticut	392	74.6	(69.1-79.5)	70.9	(65.4-75.8)	59.8	(54.0-65.4)	44.3	(38.5-50.3)
Delaware	348	73.1	(66.3-79.0)	70.8	(63.0-77.6)	58.3	(50.7-65.5)	38.8	(32.3-45.7)
District of Columbia	146	78.0	(68.4-85.3)	63.9	(54.2-72.6)	43.4	(34.0-53.4)	34.5	(25.8-44.4)
Florida	600	66.5	(61.9-70.8)	60.5	(56.0-64.8)	57.8	(53.3-62.2)	36.6	(32.4-41.1)
Georgia	290	64.9	(58.0-71.2)	60.3	(53.7-66.6)	56.5	(49.5-63.2)	33.0	(27.1-39.5)
Hawaii	485	49.3	(43.6-55.0)	74.1	(68.9-78.7)	53.3	(47.6-59.0)	33.8	(28.5-39.6)
Idaho	388	54.6	(48.9-60.2)	65.4	(59.9-70.6)	56.2	(50.5-61.7)	34.2	(28.9-39.8)
Illinois^f^	---	---	---	---	---	---	---	---	---
Indiana	388	53.5	(47.9-58.9)	68.0	(62.7-72.9)	61.4	(55.9-66.6)	33.3	(28.3-38.8)
Iowa	291	63.2	(56.7-69.2)	73.4	(67.3-78.7)	65.2	(58.6-71.2)	42.0	(35.7-48.6)
Kansas	307	64.5	(58.3-70.3)	72.6	(66.7-77.7)	64.9	(58.4-70.8)	43.2	(37.0-49.5)
Kentucky	506	58.0	(52.1-63.7)	67.9	(62.5-72.8)	56.3	(50.3-62.0)	36.3	(30.6-42.4)
Louisiana	334	57.6	(51.4-63.5)	56.4	(50.4-62.2)	58.5	(52.5-64.2)	31.0	(25.7-36.9)
Maine	161	73.1	(64.6-80.2)	73.2	(65.0-80.1)	67.7	(59.3-75.2)	50.1	(41.5-58.6)
Maryland	289	76.8	(70.3-82.3)	69.7	(62.5-76.1)	59.7	(52.0-67.0)	46.4	(38.8-54.2)
Massachusetts	499	69.0	(63.9-73.7)	70.4	(65.4-75.0)	61.7	(56.4-66.7)	43.8	(38.5-49.2)
Michigan	435	72.1	(66.8-76.8)	66.4	(60.9-71.5)	60.8	(55.2-66.2)	43.4	(37.8-49.1)
Minnesota	305	80.5	(75.3-84.9)	74.2	(68.6-79.1)	67.8	(61.7-73.3)	53.9	(47.7-60.0)
Mississippi	248	55.2	(48.3-62.0)	61.2	(54.4-67.6)	56.2	(49.3-62.9)	28.9	(23.1-35.5)
Missouri	381	52.8	(46.2-59.3)	66.2	(59.7-72.2)	57.3	(50.6-63.6)	31.1	(25.4-37.5)
Montana	311	57.2	(49.8-64.3)	65.4	(58.3-71.9)	62.1	(55.0-68.8)	35.7	(28.9-43.2)
Nebraska	393	53.9	(48.3-59.4)	63.7	(58.2-68.8)	57.9	(52.4-63.2)	32.4	(27.4-37.7)
Nevada	266	60.6	(51.9-68.7)	55.8	(47.0-64.2)	61.7	(52.7-69.9)	32.9	(25.3-41.5)
New Hampshire	331	67.9	(61.9-73.4)	73.0	(67.5-77.8)	62.9	(57.1-68.3)	39.8	(34.0-45.8)
New Jersey	491	64.7	(56.3-72.3)	74.8	(67.2-81.1)	62.7	(53.9-70.8)	39.9	(31.6-48.9)
New Mexico	410	59.4	(53.8-64.7)	67.0	(61.5-72.0)	62.4	(56.9-67.6)	38.6	(33.2-44.3)
New York	289	70.6	(64.2-76.3)	65.7	(59.2-71.7)	64.6	(57.8-70.8)	43.3	(36.7-50.2)
North Carolina	485	61.0	(54.1-67.5)	69.4	(62.8-75.3)	62.6	(55.8-68.9)	40.4	(33.4-47.7)
North Dakota	206	63.8	(56.2-70.7)	78.4	(71.5-84.0)	74.0	(66.6-80.2)	46.5	(39.1-54.2)
Ohio	262	57.3	(50.1-64.1)	68.1	(61.3-74.2)	61.2	(53.9-67.9)	34.1	(27.6-41.3)
Oklahoma	605	54.6	(50.2-58.9)	75.2	(71.4-78.7)	65.4	(61.1-69.4)	38.2	(34.0-42.6)
Oregon	213	63.8	(56.5-70.6)	65.9	(58.9-72.3)	60.5	(53.2-67.4)	34.5	(27.7-41.9)
Pennsylvania	999	64.8	(60.9-68.4)	72.0	(68.5-75.2)	62.4	(58.5-66.1)	40.2	(36.4-44.2)
Rhode Island	273	76.0	(69.8-81.2)	74.4	(68.4-79.6)	62.2	(55.8-68.1)	48.4	(41.9-55.0)
South Carolina	319	64.8	(58.0-71.0)	68.0	(61.4-73.9)	64.4	(57.6-70.8)	42.6	(35.8-49.7)
South Dakota	415	60.0	(54.8-65.1)	74.0	(69.2-78.3)	55.4	(50.0-60.6)	35.2	(30.1-40.6)
Tennessee	249	59.3	(52.3-65.9)	70.2	(63.6-76.1)	59.6	(52.6-66.2)	38.1	(31.5-45.2)
Texas	359	61.0	(55.0-66.5)	58.7	(52.9-64.3)	52.8	(46.9-58.5)	31.9	(26.7-37.5)
Utah	285	57.0	(49.9-63.8)	73.3	(66.7-79.1)	66.3	(59.3-72.7)	41.9	(35.1-48.9)
Vermont	312	74.2	(68.5-79.1)	74.5	(69.1-79.2)	64.7	(58.7-70.2)	49.9	(43.8-56.1)
Virginia	292	64.1	(55.7-71.7)	66.3	(57.9-73.7)	57.9	(49.9-65.4)	33.9	(26.8-41.9)
Washington	324	62.3	(55.9-68.3)	59.4	(53.0-65.6)	58.0	(51.5-64.3)	36.5	(30.7-42.7)
West Virginia	276	57.9	(51.5-64.1)	67.4	(61.0-73.2)	61.3	(54.8-67.4)	35.7	(29.7-42.1)
Wisconsin	282	69.8	(63.3-75.6)	75.7	(69.8-80.8)	68.5	(61.8-74.5)	45.4	(38.6-52.4)
Wyoming	251	51.5	(44.6-58.3)	70.5	(63.5-76.6)	64.6	(57.4-71.1)	33.1	(27.0-39.8)

Lowest		49.3		55.8		43.4		25.8	
Highest		80.5		78.4		74.0		53.9	
Median^g^		63.8		69.5		61.2		38.2	

a Met colorectal cancer screening recommendations (either fecal occult blood testing [FOBT] in the past year or endoscopy [colonoscopy or sigmoidoscopy] within the past 10 years).

b Met recommendation for influenza vaccination (influenza vaccination in the past year).

c Met recommendation for pneumonia vaccination (ever had pneumonia vaccination).

d Up-to-date on preventive services: met colorectal cancer screening recommendations and immunization recommendations.

e CI indicates confidence interval.

f Illinois data are not included because the state used a split sample for some preventive services questions.

g Median represents the median value among the 49 states and the District of Columbia.

### Appendix

**Table:**

	** **	**Breast Cancer Screening^a^**		**Cervical Cancer Screening^b^**		**Colon Cancer Screening^c^**		**Influenza Vaccination^d^**		**Pneumococcal Vaccination^e^**		**Up-to-Date^f^**	

	** N**	**%**	**(95% CI)^g^**	**%**	**(95% CI)**	**%**	**(95% CI)**	**%**	**(95% CI)**	**%**	**(95% CI)**	**%**	**(95% CI)**
Alabama	482	80.1	(75.7-83.9)	77.1	(70.0-82.9)	54.3	(49.2-59.3)	64.9	(59.9-69.5)	59.1	(54.1-63.9)	29.8	(25.4-34.7)
Alaska	166	76.6	(64.3-85.6)	79.4	(64.6-89.1)	66.0	(55.3-75.4)	74.7	(64.9-82.5)	70.2	(59.1-79.4)	36.8	(25.6-49.7)
Arizona	527	81.0	(76.4-84.9)	83.7	(76.6-89.0)	60.4	(54.1-66.3)	67.8	(61.9-73.2)	70.4	(64.8-75.5)	38.0	(32.0-44.4)
Arkansas	600	68.4	(64.2-72.4)	64.3	(57.6-70.5)	50.8	(46.3-55.3)	68.2	(63.9-72.3)	59.1	(54.6-63.4)	27.2	(23.3-31.4)
California	472	80.7	(74.6-85.6)	82.3	(76.0-87.3)	59.2	(52.8-65.3)	72.8	(66.8-78.0)	71.3	(65.3-76.7)	38.1	(32.3-44.2)
Colorado	432	75.2	(70.2-79.7)	73.9	(67.4-79.4)	62.8	(55.8-69.2)	70.8	(63.7-77.0)	69.4	(62.3-75.7)	34.0	(28.7-39.7)
Connecticut	708	81.9	(78.3-85.0)	76.2	(71.0-80.7)	66.4	(62.1-70.5)	71.8	(67.7-75.6)	67.7	(63.3-71.7)	39.0	(34.7-43.5)
Delaware	575	83.3	(78.9-86.9)	74.8	(68.5-80.3)	65.8	(60.7-70.6)	72.0	(67.1-76.5)	68.4	(63.5-73.0)	37.4	(32.3-42.9)
District of Columbia	259	82.8	(76.0-87.9)	73.7	(64.8-81.0)	73.9	(66.7-80.0)	55.5	(48.0-62.6)	50.6	(43.2-58.0)	31.0	(24.6-38.3)
Florida	957	81.8	(78.7-84.5)	77.0	(72.6-80.9)	67.0	(63.5-70.4)	54.3	(50.6-57.9)	56.8	(53.0-60.4)	32.4	(29.0-36.0)
Georgia	598	77.5	(72.8-81.6)	70.3	(63.1-76.6)	58.3	(53.1-63.3)	58.6	(53.6-63.5)	57.8	(52.8-62.6)	28.1	(23.8-32.8)
Hawaii	764	72.4	(67.5-76.7)	71.3	(64.9-76.9)	55.4	(50.4-60.3)	73.7	(69.4-77.5)	64.5	(59.7-69.0)	34.1	(29.6-38.8)
Idaho	688	70.3	(66.2-74.1)	63.0	(56.5-69.1)	58.1	(53.7-62.5)	64.9	(60.5-69.0)	58.4	(54.0-62.7)	29.5	(25.4-34.0)
Illinois^h^	---	---	---	---	---	---	---	---	---	---	---	---	---
Indiana	710	72.9	(69.1-76.4)	70.9	(65.6-75.7)	55.0	(50.7-59.2)	65.3	(61.1-69.2)	61.0	(56.8-65.1)	23.2	(19.8-27.0)
Iowa	630	76.7	(72.7-80.2)	75.4	(69.6-80.3)	59.7	(54.8-64.4)	73.5	(69.3-77.3)	66.9	(62.2-71.2)	34.3	(29.8-39.1)
Kansas	586	80.1	(76.4-83.4)	77.6	(72.2-82.2)	56.9	(52.3-61.4)	66.0	(61.6-70.1)	60.2	(55.6-64.6)	28.5	(24.3-33.0)
Kentucky	1315	77.2	(74.0-80.2)	76.4	(71.5-80.7)	57.7	(53.8-61.5)	64.3	(60.7-67.8)	56.8	(53.1-60.5)	26.8	(23.2-30.8)
Louisiana	692	79.6	(75.9-82.9)	74.9	(68.3-80.4)	50.9	(46.5-55.2)	57.9	(53.6-62.0)	54.8	(50.5-59.0)	23.0	(19.7-26.7)
Maine	318	82.8	(77.8-86.8)	80.7	(73.6-86.3)	63.9	(57.8-69.6)	74.2	(68.6-79.1)	66.1	(60.1-71.7)	37.8	(31.9-44.0)
Maryland	478	77.6	(72.1-82.2)	76.4	(69.3-82.2)	68.4	(62.9-73.5)	63.2	(57.4-68.7)	66.0	(60.3-71.3)	33.6	(28.2-39.4)
Massachusetts	908	82.3	(79.0-85.1)	73.4	(68.8-77.6)	65.3	(61.4-69.1)	74.0	(70.3-77.4)	64.6	(60.6-68.3)	37.4	(33.3-41.6)
Michigan	740	79.4	(75.6-82.8)	75.3	(69.9-79.9)	65.1	(60.7-69.3)	68.6	(64.4-72.6)	64.3	(59.9-68.5)	33.2	(29.0-37.6)
Minnesota	640	81.3	(77.8-84.2)	81.5	(77.1-85.3)	74.6	(70.8-78.1)	78.3	(74.7-81.6)	72.2	(68.2-75.8)	46.3	(41.9-50.8)
Mississippi	647	69.5	(65.3-73.3)	61.2	(54.3-67.6)	56.4	(51.9-60.8)	64.2	(59.8-68.4)	60.7	(56.2-65.0)	27.4	(23.5-31.7)
Missouri	761	72.6	(68.0-76.8)	65.4	(58.9-71.3)	57.0	(51.9-62.0)	70.3	(65.3-74.8)	63.2	(58.0-68.1)	29.4	(24.9-34.3)
Montana	537	73.8	(68.5-78.4)	78.7	(71.8-84.3)	63.2	(57.6-68.4)	69.4	(64.0-74.4)	71.2	(65.7-76.1)	39.7	(34.1-45.6)
Nebraska	754	73.9	(70.3-77.1)	71.7	(66.6-76.2)	53.3	(49.2-57.3)	71.4	(67.7-74.9)	63.6	(59.7-67.3)	26.7	(23.0-30.7)
Nevada	337	76.9	(69.2-83.2)	66.7	(55.1-76.6)	60.3	(52.4-67.6)	64.3	(56.7-71.2)	67.7	(59.8-74.6)	34.2	(27.5-41.6)
New Hampshire	562	79.7	(75.5-83.4)	78.3	(72.7-83.0)	64.9	(60.2-69.3)	71.9	(67.6-75.8)	64.4	(59.9-68.7)	34.2	(29.9-38.9)
New Jersey	813	74.8	(69.0-79.9)	70.5	(62.7-77.3)	63.5	(56.9-69.7)	65.4	(58.8-71.4)	63.4	(56.7-69.7)	33.3	(26.8-40.4)
New Mexico	625	71.9	(67.6-75.9)	68.0	(61.7-73.7)	52.0	(47.1-56.8)	66.4	(61.7-70.7)	63.0	(58.4-67.3)	26.7	(22.4-31.6)
New York	532	77.7	(73.2-81.6)	72.4	(66.3-77.7)	62.9	(57.7-67.7)	63.9	(58.9-68.7)	61.1	(55.8-66.0)	28.0	(23.8-32.5)
North Carolina	944	81.1	(77.3-84.4)	79.5	(73.9-84.2)	59.8	(54.9-64.5)	67.3	(62.7-71.7)	63.2	(58.4-67.8)	34.0	(29.5-38.7)
North Dakota	439	78.7	(74.2-82.5)	78.8	(72.6-83.9)	64.1	(58.9-69.0)	70.7	(65.8-75.2)	71.5	(66.7-75.9)	37.7	(32.3-43.4)
Ohio	513	77.1	(72.6-81.1)	73.8	(66.8-79.8)	56.4	(50.3-62.3)	65.7	(59.5-71.4)	65.3	(59.0-71.0)	29.5	(24.7-34.9)
Oklahoma	1,083	68.4	(65.2-71.5)	64.9	(59.7-69.7)	50.9	(47.5-54.3)	70.9	(67.6-73.9)	65.6	(62.2-68.9)	25.7	(22.8-28.8)
Oregon	408	83.0	(78.7-86.5)	73.0	(65.7-79.1)	68.3	(63.1-73.1)	69.6	(64.6-74.2)	68.1	(63.0-72.9)	39.1	(33.8-44.7)
Pennsylvania	1,985	77.0	(74.6-79.3)	68.4	(64.7-71.9)	58.8	(56.0-61.6)	69.6	(66.9-72.1)	64.3	(61.5-67.0)	32.6	(29.9-35.5)
Rhode Island	477	85.8	(82.2-88.8)	74.7	(68.7-80.0)	66.2	(61.2-70.8)	73.2	(68.5-77.4)	71.1	(66.3-75.4)	37.9	(33.1-43.1)
South Carolina	583	80.5	(75.9-84.4)	87.5	(82.3-91.3)	64.1	(58.9-69.0)	70.3	(65.3-74.9)	65.1	(59.9-70.0)	36.1	(31.0-41.5)
South Dakota	775	76.5	(73.0-79.6)	71.8	(66.6-76.5)	54.5	(50.6-58.4)	74.3	(70.5-77.8)	57.6	(53.7-61.5)	26.5	(23.3-30.1)
Tennessee	423	74.3	(69.3-78.7)	71.2	(63.5-77.8)	58.6	(53.3-63.8)	72.6	(67.7-77.0)	62.6	(57.4-67.5)	30.3	(25.5-35.6)
Texas	672	70.6	(66.0-74.8)	70.2	(62.7-76.7)	56.9	(52.1-61.5)	62.6	(57.8-67.2)	59.8	(55.0-64.4)	27.1	(23.2-31.4)
Utah	403	70.8	(64.3-76.5)	61.5	(50.8-71.1)	57.6	(50.9-64.1)	69.3	(62.7-75.2)	63.9	(57.1-70.2)	27.8	(22.2-34.1)
Vermont	509	78.9	(74.9-82.5)	76.5	(71.2-81.1)	65.1	(60.4-69.5)	73.0	(68.6-77.0)	67.4	(62.7-71.7)	35.0	(30.4-39.9)
Virginia	513	76.7	(72.1-80.8)	79.7	(72.7-85.2)	59.5	(54.0-64.7)	64.6	(59.4-69.5)	62.8	(57.5-67.8)	32.5	(27.7-37.8)
Washington	597	76.8	(72.1-80.9)	76.2	(70.0-81.4)	68.8	(64.0-73.3)	69.2	(64.6-73.4)	66.5	(61.4-71.2)	36.4	(31.6-41.6)
West Virginia	570	69.3	(65.0-73.2)	67.5	(61.7-72.8)	48.5	(43.9-53.0)	64.7	(60.2-68.9)	61.1	(56.6-65.4)	24.4	(20.6-28.6)
Wisconsin	565	80.3	(76.3-83.7)	74.6	(68.6-79.7)	66.3	(61.4-70.8)	72.8	(68.3-76.8)	72.0	(67.5-76.0)	38.7	(33.9-43.6)
Wyoming	444	69.0	(64.1-73.5)	67.8	(60.8-74.1)	54.0	(49.0-59.0)	70.7	(66.0-75.1)	71.0	(66.2-75.3)	30.0	(25.5-34.9)

Lowest		68.4		61.2		48.5		54.3		50.6		23.0	
Highest		85.8		87.5		74.6		78.3		72.2		46.3	
Median^i^		77.2		74.2		59.7		69.3		64.4		32.9	

a Met breast cancer screening recommendations (mammogram within 2 years).

b Met cervical cancer screening recommendations (Pap test in the past 3 years, unless the respondent had a hysterectomy and a Pap test was not needed).

c Met colorectal cancer screening recommendations (either fecal occult blood testing [FOBT] in the past year or endoscopy [colonoscopy or sigmoidoscopy] within the past 10 years).

d Met influenza recommendation (influenza vaccination in the past year).

e Met recommendation for pneumococcal vaccination (ever had pneumonia vaccination).

f Up-to-date on preventive services: met screening recommendations for breast cancer, cervical cancer (or if the respondent had a hysterectomy and cervical cancer screening was not recommended), colorectal cancer, and immunizations.

g CI indicates confidence interval.

h Illinois data are not included because the state used a split sample for some preventive services questions.

i Median represents the median value among the 49 states and the District of Columbia.
